# Genome Assembly for a Yunnan-Guizhou Plateau “3E” Fish, *Anabarilius grahami* (Regan), and Its Evolutionary and Genetic Applications

**DOI:** 10.3389/fgene.2018.00614

**Published:** 2018-12-04

**Authors:** Wansheng Jiang, Ying Qiu, Xiaofu Pan, Yuanwei Zhang, Xiaoai Wang, Yunyun Lv, Chao Bian, Jia Li, Xinxin You, Jieming Chen, Kunfeng Yang, Jinlong Yang, Chao Sun, Qian Liu, Le Cheng, Junxing Yang, Qiong Shi

**Affiliations:** ^1^State Key Laboratory of Genetic Resources and Evolution, Kunming Institute of Zoology, Chinese Academy of Sciences, Kunming, China; ^2^BGI Education Center, University of Chinese Academy of Sciences, Shenzhen, China; ^3^Shenzhen Key Lab of Marine Genomics, Guangdong Provincial Key Lab of Molecular Breeding in Marine Economic Animals, BGI Academy of Marine Sciences, BGI Marine, BGI, Shenzhen, China; ^4^BGI-Yunnan, BGI-Shenzhen, Kunming, China

**Keywords:** genome sequencing, population history, SSR, plateau fish, Cyprinidae

## Abstract

A Yunnan-Guizhou Plateau fish, the Kanglang white minnow (*Anabarilius grahami*), is a typical “3E” (Endangered, Endemic, and Economic) species in China. Its distribution is limited to Fuxian Lake, the nation’s second deepest lake, with a significant local economic value but a drastically declining wild population. This species has been evaluated as VU (Vulnerable) in the China Species Red List. As one of the “Four Famous Fish” in Yunnan province, the artificial breeding has been achieved since 2003. It has not only re-established its wild natural populations by reintroduction of the artificial breeding stocks, but also brought a wide and popular utilization of this species to the local fish farms. *A. grahami* has become one of the main native aquaculture species in Yunnan province, and the artificial production has been emerging in steady growth each year. To promote the conservation and sustainable utilization of this fish, we initiated its whole genome sequencing project using an Illumina Hiseq2500 platform. The assembled genome size of *A. grahami* is 1.006 Gb, accounting for 98.63% of the estimated genome size (1.020 Gb), with contig N50 and scaffold N50 values of 26.4 kb and 4.41 Mb, respectively. Approximately about 50.38% of the genome was repetitive. A total of 25,520 protein-coding genes were subsequently predicted. A phylogenetic tree based on 4,580 single-copy genes from *A. grahami* and 18 other cyprinids revealed three well-supported subclades within the Cyprinidae. This is the first inter-subfamily relationship of cyprinids at genome level, providing a simple yet useful framework for understanding the traditional but popular subfamily classification systems. Interestingly, a further population demography of *A. grahami* uncovered a historical relationship between this fish and Fuxian Lake, suggesting that range expansion or shrinkage of the habitat has had a remarkable impact on the population size of endemic plateau fishes. Additionally, a total of 33,836 simple sequence repeats (SSR) markers were identified, and 11 loci were evaluated for a preliminary genetic diversity analysis in this study, thus providing another useful genetic resource for studying this “3E” species.

## Introduction

The Yunnan-Guizhou Plateau (or Yungui Plateau) is a highland region primarily located in the Yunnan and Guizhou provinces in the southwest part of China. This mountain area harbors large numbers of plants and terrestrial vertebrates, and contains 4 of the 10 hotspot ecoregions in China (the Xishuangbanna area, and the Hengduan, Wumeng, and Wuling mountains, Tang et al., 2006). It also holds an abundance of aquatic species, as it encompasses the headwaters of many of the great rivers in Asia that originate on the Qinghai-Tibet Plateau (e.g., the Salween, Mekong and Yangtze rivers). As a consequence, Yunnan province possesses the greatest diversity of fishes in China, accounting for 40% of the nation’s freshwater fish species (Chen, 2013).

Most of the native fishes in Yunnan province are locally endemic. The Kanglang white minnow (*Anabarilius grahami*) is but one example. It is a cyprinoid fish with restricted distribution in Fuxian Lake, a typical Yunnan-Guizhou plateau lake and also the second deepest lake in China. The species is one of the “Four Famous Fish” in Yunnan that has a special value and popularity. Although it is a small-sized fish, it has long been the major economic fish species in Fuxian Lake, accounting for 70–80% of the natural fishery production before 1990s (Li et al., 2003a). This fish is historically famous because of its good taste and flavor – attribute to its special muscle nutrition compositions (Deng et al., 2013) – as well as some folkloric medicinal functions and in appealing to fishing cultures. Along with the long-term formation of Fuxian Lake, *A. grahami* has many special biological characters that were thought to be a result of adaptation of the fish to the lake (Yang, 1992). For instance, because of the limited food resources in the oligotrophic Fuxian Lake, it has a very low absolute fecundity (number of mature eggs: 2,175–3,840) relative to its sister species, the *Anabarilius andersoni* (13,971–15,770) in the adjacent Xinyun Lake (Yang, 1992). As a way of compensating, it has a long annual breeding period from March to October, and shows unusual spawning behaviors, such as a temporally regular interval (*ca*. 7 days) between two sequentially spawnings (Yang, 1992; Ma et al., 2008). It would also be an adaptation to the limited spawning sites that are only available at some cave or hill springs around the Fuxian Lake. In addition, the larvae and adults of *A. grahami* occupy distinct habitats, with the larvae and juveniles occurring in the shallow coastal regions and the adults in the middle and upper layers of open water (Yang, 1992; Ma et al., 2008). This seems to be a response to the limited food resources in the whole lake (Yang, 1994). As the second deepest lake of China, Fuxian Lake has a relatively broad niche in terms of water depth. Correspondingly, the adults of *A. grahami* can frequently be found in water depths down to 20 m, and may occasionally be seen as deep as 50 m (Yang, 1992). Thus *A. grahami* is unusual among other species of the Cultrinae, because most of them are thought to live in the upper to middle levels (probably less them 5 m) of shallow lakes or rivers (Chen, 1998). The spatial dichotomy strategy of *A. grahami* might be also a crucial reason enabling it to maintain the largest natural fish stocks in Fuxian Lake. However, all of the interesting biological questions on *A. grahami* are hypothetical, and have not been empirically explored.

In recent decades, however, the wild population of *A. grahami* has decreased sharply. It has been triggered by the introduction in 1982 of the exotic icefish, *Neosalanx taihuensis*. The annual production of *A. grahami* declined from about 400 tons before the 1990s, to 10.4 tons in the 1998, and finally to less than 1 ton in the early 2000s; while the annual production of *N. taihuensis* has increased since 1990s, from about 200 tons during the early colonized years (1986–1990) to an average of 1,554 tons during 1990–2004 (Xiong et al., 2006). Competitive disadvantage has been ascribed for the population decline of *A. grahami*, because the exotic *N. taihuensis* and the endemic *A. grahami* have significant food and space overlaps (Qin et al., 2007). However, other anthropogenic causes, such as overfishing of *A. grahami*, destruction of the spawning sites, and the collateral damage by catching *N. taihuensis*, should also be considered (Li et al., 2003a). At the same time, the low fecundity of *A. grahami* itself (Yang, 1992) might also make it vulnerable in the changing environment. The drastic population decline of *A. grahami* shifted it from an abundant economic species to an endangered fish. This valuable fish was evaluated as VU (vulnerable) in the China Species Red List in 2004 (Wang and Xie, 2004) and 2015 (Jiang et al., 2016), and among the threatened fishes of the world (Liu et al., 2009). Fortunately, artificial breeding was achieved in 2003 (Li et al., 2003b), and reintroduction of the breeding stocks has become almost the only way to re-establish its wild populations. However, the adaptability and sustainability of the re-established wild population, as well as the current genetic diversity (after serious population fluctuation) are unexplored areas that await evaluation; lack of effective genetic markers might be one reason for this situation. On the other hand, artificial breeding has also created the chance of aquaculture utilization of this valuable species. Although the artificial cultivation is still a small-scale operation, the annual production has gradually increased since 2005, and reached about 15 tons in 2014 from the fish farms around Fuxian Lake (Li, 2015). At present, *A. grahami* has been one of the main native aquaculture species in Yunnan province, and the utilization of this species in local aquaculture has been exhibiting in steady growth each year.

*Anabarilius grahami* is a typical species with “3E” (Endangered, Endemic, and Economic) status and priorities. We therefore initiated the whole genome sequencing (WGS) project of this valuable species. The WGS would promote the aspect of many biological and conservational enquiries, and also provide extensive opportunities for its utilization in aquaculture. Based on the WGS information, we also aimed to carry out three evolutionary and genetic applications in this study: (1) reconstruction of the inter-subfamily phylogenetic relationship within the Cyprinidae from a genomic view, (2) reconstruction of the demographic history of *A. grahami* along with the formation of Fuxian Lake, and (3) development of massive simple sequence repeats (SSR) markers for the future genetic evaluation of this “3E” plateau fish species.

## Materials and Methods

### Sample Preparation and Genome Sequencing

Samples of *A. grahami* were collected from artificial cultivated stocks in the Endangered Fish Conservation Center (EFCC) of the Kunming Institute of Zoology, Chinese Academy of Sciences (KIZ), Kunming, China. The research protocol and treatment of experimental fishes was reviewed and approved by the internal review board of KIZ (approval ID: 2015-SMKX026).

Genomic DNA was extracted from a pool of muscle tissue from two individuals. Three short paired-end (200, 500, and 800 bp) and four long paired-end (2, 5, 10, and 20 kb, respectively) sequencing libraries were constructed with the standard protocol provided by Illumina (San Diego, United States), and then sequenced on an Illumina Hiseq2500 platform. Low-quality and duplicated reads were filtered out through SOAPfilter (v2.2) software (Li R. et al., 2009).

For transcriptome-based prediction, RNA was extracted from four tissues (brain, liver, gonad and muscle) of the same two individuals. All the libraries were prepared using the Illumina TruSeq RNA sample preparation kit (San Diego, United States) and then sequenced by Illumina Hiseq4000.

### Genome Assembly

The genome size was estimated using the 17-mer depth frequency distribution formula (Liu et al., 2013) as follows: G (Genome size) = k-mer_number/k-mer_depth, where k-mer_number is the total number of k-mer, and k-mer_depth indicates the peak frequency that is higher than others. The clean reads were used to construct contigs and original scaffolds by assembler, Platanus (v1.2.4, Kajitani et al., 2014) with default parameters. Subsequently, intra-scaffold gaps were filled using the reads of short-insert libraries by GapCloser 1.12 (Li R. et al., 2009). BUSCO (Benchmarking Universal Single-Copy Orthologs; v3.0.2, Simao et al., 2015) was employed to evaluate the completeness of achieved genome assembly.

### Genome Annotation

We identified repetitive sequences using the following pipeline. At first, Tandem Repeats Finder (v4.07, Benson, 1999) was used to search tandem repeats in the genome assembly. Subsequently, we combined both homology-based and *de novo* predictions to identify transposable elements (TEs). We utilized RepeatMask (v1.323, Tarailo-Graovac and Chen, 2009) to detect known TEs against the Repbase TE library (release 21.01, Jurka et al., 2005) and RepeatProteinMask (v2.1) to identify the TE correlated proteins. Subsequently, we used LTR_FINDER (Xu and Wang, 2007) and RepeatModeler (v1.73, Abrusan et al., 2009) to construct a *de novo* repeat library with default parameters. Finally, we employed RepeatMask (Tarailo-Graovac and Chen, 2009) to identify known and novel TEs against Repbase TE and the *de novo* repeat library.

We combined *de novo*, homology-based and transcriptome-based prediction methods to predict protein-coding genes. For the *de novo* prediction, AUGUSTUS 3.0.1 (Stanke et al., 2006) and GenScan 1.0 (Burge and Karlin, 1997) were employed to predict gene structures of the repeat-masked genome assembly. For the homology-based prediction, the reference protein sequences were from five fishes, including zebrafish (*Danio rerio*, Howe et al., 2013), medaka (*Oryzias latipes*, Kasahara et al., 2007), a Chinese cavefish (*Sinocyclocheilus grahami*, Yang et al., 2016), grass carp (*Ctenopharyngodon idella*, Wang et al., 2015) and common carp (*Cyprinus carpio*, Xu et al., 2014). These downloaded protein sequences were mapped onto the assembled genome using tBlastn (v22.19, Mount, 2007) with *E*-value threshold of 1*e*-5. Genewise (v2.2.0) was employed to predict gene structures. The RNA-Seq data were aligned to the genome assembly using TopHat (v2.0, Trapnell et al., 2009) and searched transcript structures with Cufflinks (Trapnell et al., 2010). Finally, all gene models from the above three methods were integrated to form a comprehensive and non-redundant gene set using GLEAN (Elsik et al., 2007).

### Functional Assignment

All protein sequences from the GLEAN results were aligned to TrEMBL and SwissProt databases (Boeckmann et al., 2003) using BlastP at *E*-value ≤ 1*e*-5. The gene pathways were mapped to the KEGG database (Kanehisa and Goto, 2000). We also used the InterProScan software (Hunter et al., 2009) to annotate the protein sequences by searching publically available databases including Pfam (Finn et al., 2014), PRINTS (Attwood, 2002), PANTHER (Thomas et al., 2003), ProDom (Bru et al., 2005) and SMART (Letunic et al., 2004). In summary, approximately 87.08% of the genes were supported by at least one related function assignments from the public databases (TrEMBL, SwissProt, KEGG and InterPro).

### Phylogenetic Analysis

To understand the phylogenetic status of *A. grahami* within the Cyprinidae, we reconstructed a phylogenetic tree with *A. grahami* and other 18 cyprinids, using channel catfish (*Ictalurus punctatus*) as the out group. These selected species covered 11 of the 12 broadly recognized subfamilies in Cyprinidae (Chen, 1998); however, no data of the remainder Gobiobotinae are available yet. Among these cyprinid species (Table 1), if the whole-genome gene sets were available, we directly adopted them; if only transcriptome data were available, we downloaded the submitted reads from NCBI and employed a *de-novo* assembled method to assembly them into gene sets. Generally, each single-copy gene in diploid species would have two corresponding copies in tetraploid genomes. We therefore randomly separated the two copies into two gene sets and then combined each of the gene sets in tetraploid species with the single gene set in diploid species to produce two final single-copy datasets (dataset I and II).

**Table 1:** Fish species selected for the phylogenetic analysis of Cyprinidae in the present study.

No.	Scientific name	Subfamily classification^∗^	Data type	Accession No.^#^
1	*Hypophthalmichthys molitrix*	Hypophthalmichthyinae	Transcriptome	SRR342398
2	*Hypophthalmichthys nobilis*	Hypophthalmichthyinae	Transcriptome	SRR3036336
3	*Microphysogobio brevirostris*	Gobioninae	Transcriptome	SRR1185341
4	*Gobio acutipinnatus*	Gobioninae	Transcriptome	SRR1660441
5	*Xenocypris argentea*	Xenocyprinae	Transcriptome	SRR5351748
6	*Culter ilishaeformis*	Cultrinae	Transcriptome	SRR959086
7	*Anabarilius grahami*	Cultrinae	Genome	**PRJNA477399**
8	*Ctenopharyngodon idella*	Leuciscinae	Genome	PRJEB5920
9	*Tinca tinca*	Leuciscinae	Transcriptome	SRR5997852
10	*Danio rerio*	Danioninae	Genome	PRJNA11776
11	*Danio albolineatus*	Danioninae	Transcriptome	SRR5451065
12	*Gymnodiptychus pachycheilus*	Schizothoracinae	Transcriptome	SRR1583887
13	*Schizothorax richardsonii*	Schizothoracinae	Transcriptome	SRR1552917
14	*Cyprinus carpio*	Cyprininae	Genome	PRJNA202478
15	*Carassius auratus*	Cyprininae	Transcriptome	SRR1038441
16	*Sinocyclocheilus grahami*	Barbinae	Genome	PRJNA274017
17	*Sinocyclocheilus anshuiensis*	Barbinae	Genome	PRJNA274017
18	*Labeo rohita*	Labeoninae	Transcriptome	SRP012989
19	*Rhodeus uyekii*	Acheilognathinae	Transcriptome	SRR2043486


*^∗^The classification of subfamily was adopted from a previous report (Chen, 1998). ^#^The accession numbers included the NCBI BioProject ID for the genome data and Sequence Read Archive (SRA) Run ID for the transcriptome data. Please note that the ID highlighted in bold referred to the genome data of *A. grahami* that we assembled in this study.*

In dataset I and II, 229 single-copy families including 4,580 single-copy genes were collected; however, the alignment yielded 247,500 and 256,839 sites, respectively. These two datasets were subsequently employed to construct phylogenetic trees using both maximum likelihood (ML) method in PhyML (v3.0, Guindon and Gascuel, 2003) and Bayes Inference (BI) method in Mrbayes (v3.1, Ronquist and Huelsenbeck, 2003).

### Heterozygous SNP Calling and Demographic History

Firstly, we identified heterozygous single-nucleotide polymorphisms (SNPs) in the *A. grahami* genome. We mapped 500-bp insert-sized reads against our assembled genome with BWA (v0.7.12-r1039, Li and Durbin, 2009). The SNPs were called by SAMtools (v0.1.19, Li H. et al., 2009) and filtered by read depth across the genome. In total, approximately 1,733,343 heterozygous sites were identified and the diploid consensus genome sequences were generated by these SNPs. Secondly, the distribution of the time since the most recent common ancestor (TMRCA) between two alleles in an individual was used to predict the history of change in population size. We employed the pairwise sequentially Markovian coalescent (PSMC) model (Li and Durbin, 2011) on heterozygous sites of *A. grahami* genome with the putative generation time (*g* = 2 years) and the mutation rate (μ = 3.51 × 10^-9^ per year per nucleotide, Graur and Li, 2000) to estimate historical effective population sizes over a range from 10^4^ to 10^7^ years ago. Finally, we used gnuplot4.4 (Janert, 2010) to draw a curve for the reconstructed population history.

### SSR Searching and Identification

We searched for SSR loci with motifs ranging from di- to hexa-nucleotides in the assembled genome scaffolds of *A. grahami*. Our mining criteria included (i) scaffolds extracted with the length ≥1 kb and the average sequence coverage >20×; (ii) SSR identified from the selected scaffolds using MISA script^1^ with default settings at (2/6) (3/5) (4/5) (5/5) (6/5), and >100 bp between two SSRs; (iii) repeat motifs and the 200-bp flanking sequences used for Blastn search against the genome sequence with *E*-value ≤ 1*e*-5; (iv) SSR developed through filtering with >90% identity and >85% alignment length of the flanking sequences; (v) final SSR loci identified as candidates for marker development with single hit when mapped back to the genome.

### SSR Evaluation and Genetic Diversity Analysis

Three steps were conducted in evaluating the efficiencies of SSR development in this study, and a preliminary genetic diversity of *A. grahami* was also assessed based on the final optimized SSR markers. We named the three steps as polymerase chain reactions (PCRs), polymorphism and parameters evaluation, respectively. Firstly, we selected a random set of 50 SSR loci for primer design using PRIMER3 (Koressaar and Remm, 2007), with expected PCR products ranging from 100 to 200 bp. Amplification effectiveness was tested on two geographically separated individuals of *A. grahami*. Secondly, we chose those good loci with correct and bright electrophoretic bands for a polymorphism evaluation, which was realized from 7 populations with three individuals in each population. Those SSR loci without any polymorphism among all the 21 samples were discarded. Thirdly, we filtered the SSR loci by means of an evaluation of the parameters that could affect the reliability of SSR analysis. This evaluation included null allele detection and linkage disequilibrium tests, based on the genotyping data matrix from four populations (30 samples in each population). These four populations, named EFCC1, EFCC2, Huoyanshan and Luchong, respectively, were artificially preserved populations from three different fish breeding farms. They were the main sources for artificial reintroduction each year to the current wild population in Fuxian Lake.

Polymerase chain reaction in the first step were carried out in 12.5-μL reaction volumes using the amplification profile as follows: 4 min at 94°C, 35 cycles of 30 s at 94°C, 35 s at 57°C, 40 s at 72°C, followed by a final extension step of 10 min at 72°C. The PCR procedures in the second and third steps were performed with the same conditions as those in the first step, except using fluorescent labeled reverse primers (6-FAM, HEX) instead of the regular primers, and using 1:(40–100) dilution of the first PCR product as the DNA template according to the brightness relative to the standard DNA marker-referred electrophoretic stripes. All PCR products were then genotyped on an ABI 3730xl genetic analyzer with Gene-Scan LIZ-500 (Applied Biosystems, United States) as the internal size standard, and scored with GeneMarker (SoftGenetics, United States). Genotyping errors associated with SSR analysis such as stutter bands, large allele dropout and null alleles were detected using MICRO-CHECKER (v2.2.3, Van Oosterhout et al., 2004). CERVUS (v3.0.7, Kalinowski et al., 2007) was employed to find matching pairs of genotypes and calculate the basic genetic parameters, including number of alleles (*Na*), polymorphism information content (*PIC*), the observed and expected heterozygosities (*Ho* and *He*), and null allele frequencies. The inbreeding coefficient (*Fis*), deviations from Hardy-Weinberg equilibrium (*HWE*), and linkage disequilibrium tests were performed with GENEPOP (v4.7.0, Rousset, 2008).

## Results

### Summary of Genome Assembly and Annotation for *A. grahami*

**FIGURE 1:**
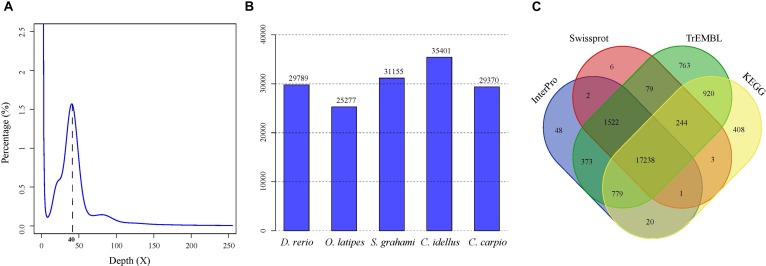
Genome-size estimation and genome annotation of *A. grahami*. **(A)** 17-mer frequency distribution of sequenced reads. **(B)** The number of predicted genes in *A. grahami* reciprocally homologous to other five representative fishes. **(C)** 22,406 predicted protein-coding genes with matching entries in the four popular public databases.

A total of 279.6-Gb raw data were generated by sequencing seven libraries on the Illumina HiSeq 2500 platform (Supplementary Table S1). The k-mer depth distributes with a main peak at 40× (Figure 1A), and therefore the genome size of *A. grahami* was estimated to be 1.020 Gb (Table 2). In addition, a minor curve at the right tail showed a low level of possible repetitive sequences (Figure 1A). After filtering low-quality reads, 188.9 Gb of clean reads were assembled using Platanus (Supplementary Table S2). The final assembled genome size of *A. grahami* is 1.006 Gb, accounting for 98.63% of the estimated genome size (1.020 Gb). The assembled contig number is 250,527 and the scaffold number is 178,229, with contig N50 and scaffold N50 values of 26.4 kb and 4.41 Mb, respectively (Table 2). The length of scaffold N50 of *A. grahami* is greater than these in all fishes but grass carp within Cyprinidae with published genomes, and also greater than these in most other non-cyprinid teleosts (see more details in Supplementary Table S3).

**Table 2:** Summary of the genome assembly and annotation for *A. grahami*.

Genome assembly	Parameter	Genome annotation	Parameter
Contig N50 size (kb)	26.37	Protein-coding gene	25,520
Scaffold N50 size (Mb)	4.41	Annotated functional gene	22,406 (87.80%)
Estimated genome size (Gb)	1.020	Unannotated functional gene	3,114 (12.20%)
Assembled genome size (Gb)	1.006	Repeat content	50.38%
Genome coverage (×)	188.88	Average gene length (bp)	9,152
Longest scaffold (bp)	18,552,664	Average exon length (bp)	197


Using BUSCO software, we chose the single-copy orthologs (*N* = 4,584) obtained from the phylogenetic analysis to assess the completeness of our genome assembly. The result showed that 93.2% of BUSCO genes were complete, in which 89.6% were single-copy BUSCOs while 3.6% were duplicated BUSCOs; 4.0% were fragmental BUSCOs, and 2.8% were missing. These data confirmed that our assembled genome was comparatively high quality and complete.

The genome comprised approximately 50.38% repetitive sequences (Table 2), which was comparable to the repeat content (52.2%) of the zebrafish genome (Howe et al., 2013, Supplementary Table S4). Additionally, the most abundant type of TE was class II DNA transposon (31.37%; Supplementary Table S5).

The number of predicted genes in *A. grahami*, reciprocally homologous to five representative fish genomes (*D. rerio*, *O. latipes*, *S. grahami*, *C. idella* and *C. carpio*), was more than 25,000 (Figure 1B). With a combination of *de novo*, homology-based and transcriptome-based annotation methods, we finally predicted a total of 25,520 protein-coding genes from the present *A. grahami* genome assembly, and 22,406 (87.80%) genes matched entries in the public databases (TrEMBL, SwissProt, KEGG and InterPro, Table 2 and Figure 1C). The total number of protein-coding genes identified in *A. grahami* (25,520) was similar to the sequenced diploid cyprinids, such as zebrafish (26,000, Howe et al., 2013) and grass carp (27,263, Wang et al., 2015), and approximately half of the tetraploid cyprinids, such as common carp (52,610, Xu et al., 2014) and the golden-line barbel fish (42,109, Yang et al., 2016). These data provided evidence to support the diploid nature of *A. grahami* from a genomic view.

### Phylogenetic Position and Population History of *A. grahami*

**FIGURE 2:**
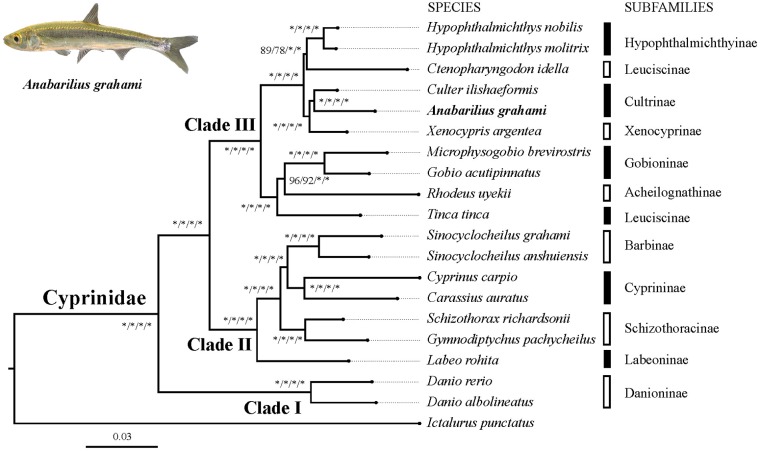
Inter-subfamily phylogenetic relationships within the Cyprinidae. The analysis was based on the 4,580 single-copy genes of two datasets (dataset I and II) using the ML and BI methods. Supporting values are presented as ML-I/ML-II/BI-I/BI-II at each node, where asterisks (^∗^) denote bootstrap value (ML) or posterior probability (BI) of 100%. The position of *A. grahami* is marked in bold, and a photo of a live specimen of this species is shown on the top left.

Based on two datasets (dataset I and II) and two methods (ML and BI), four phylogenetic trees (ML-I, ML-II, BI-I and BI-II) were obtained. All the four trees revealed an identical topology of the 19 species in the Cyrpinidae involved in the study, representing 11 of the 12 recognized subfamilies (Chen, 1998; Figure 2). Within this group, the closest relative of *A. grahami* is *Culter ilishaedormis*; both of them belong to the subfamily of Cultrinae. The 11 subfamilies were all recovered as monophyletic groups except Leuciscinae, in which the *Tinca tinca* was not nested with *C. idella*, but had a closer relationship with the species representing the Gobioninae and the Acheilognathinae.

Three major subclades (Clade I, II and III) were recovered in the Cyprinidae with strong supporting values (Figure 2). In summary, the Clade I represented the subfamily Danioninae, which was resolved as the basal-most subfamily within the Cyprinidae. The Clade II was recovered in a relationship of [Labeoninae, (Schizothoracinae, (Cyprininae, Barbinae))], and the Clade III was recovered in a relationship of [(*Tinca*, (Acheilognathinae, Gobioninae)), ((Leuciscinae, Hypophthalmichthyinae), (Xenocyprinae, Cultrinae))].

**FIGURE 3:**
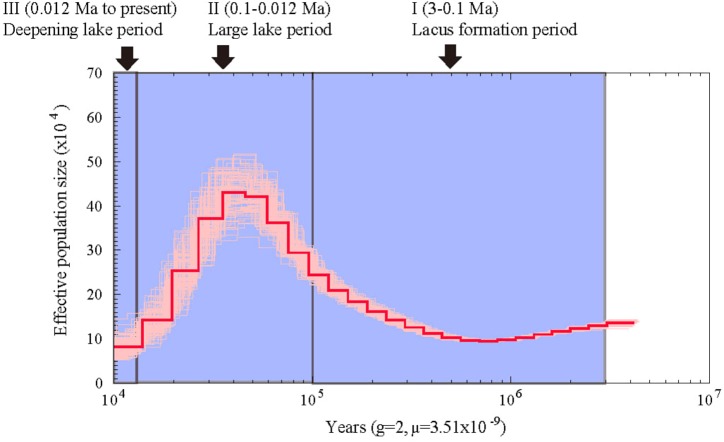
Estimated population demography of *A. grahami* using the PSMC model. The bold red line represents the estimated effective population size changes of *A. grahami*, and the thin pink lines represent 100 bootstrap estimations. The demarcated blue blocks denote three main periods during the development of Fuxian Lake, including (I) lacus formation period (3–0.1 Ma), (II) large lake period (0.1–0.012 Ma), and (III) deepening lake period (0.012 Ma to present).

Using the heterozygous SNPs from the genome data of *A. grahami*, we reconstructed the population demography based on the PSMC model. As shown in Figure 3, the population of *A. grahami* had been maintaining in a relative stable size for a long time (0.6–3 Ma), then increasing since 0.6 Ma, reaching to a peak at about 0.03–0.04 Ma, and then declining in the subsequent phase.

### SSR Identification, Evaluation and Application

**FIGURE 4:**
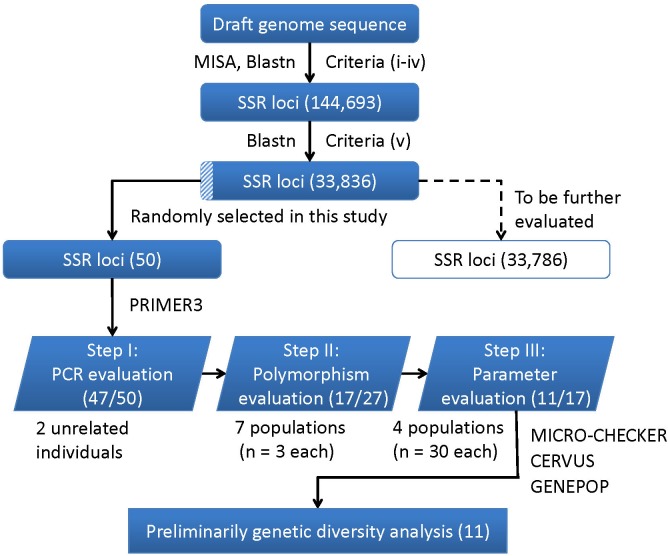
A flowchart for the process of SSR loci identification, evaluation, and application in this study. The corresponding numbers of loci retained after each step are presented in brackets.

A flowchart depicting the process used for SSR markers identification, evaluation and application is presented in Figure 4. In brief, a total of 144,693 SSR were developed using the criteria from (i) to (iv), and 33,836 were identified as final SSR loci after (v) (Supplementary Table S6). The numbers of both the developed SSR loci (144,693) and those finally identified loci (33,836) gradually decreased from di- to hexa-nucleotides motifs, while the di- plus tri-nucleotide SSRs accounted for over 98% of all the final identified SSR loci (Supplementary Table S7). For the 50 randomly selected SSR loci, 47 loci (94%) were successfully amplified PCR products with a single band and expected size (Step I: PCR evaluation). Using 27 SSR loci (22 di-, 4 tri-, and 1 tetra-nucleotide SSRs) in seven different populations (*n* = 3 in each population), only 17 of the 22 di-nucleotide loci were detected with SSR polymorphism (Step II: polymorphism evaluation), and thus retained to the next step. After excluding three loci with detection of null alleles (using MICRO-CHECK), two loci with null allele frequency greater than 0.2 (using CERVUS), two loci involved in linkage disequilibrium (using GENPOP, Supplementary Table S8), only 11 loci were finally retained (Step III: parameters evaluation). These 11 optimized SSR markers (Supplementary Table S9) were then used for a subsequent genetic diversity analysis.

Basic genetic parameters of four different populations in *A. grahami* based on the 11 SSR markers are summarized in Table 3 (more details in Supplementary Table S10). In all the four populations, the mean *Ho* (0.391∼0.467) was higher than the mean *He* (0.354∼0.411). The *PIC* values decreased in the order EFCC2 > Huoyanshan > EFCC2 > Luchong, and the average value was about 0.3 among all four populations, which indicated a moderate polymorphism in *A. grahami*. The majority of the *Fis* values were negative, indicating that the inbreeding level was relatively low. Based on the 11 SSR markers, significant deviation from *HWE* was observed only in the EFCC2 population (*P* < 0.05), and the heterozygosity excess may contribute somehow as *Ho* was relatively higher than *He* in this population.

**Table 3:** The average genetic parameters at 11 SSR loci of *A. grahami* in four different populations (*n* = 30 per population).

Populations	EFCC1		EFCC2		Huoyanshan		Luchong	
				
	Mean	SD	Mean	SD	Mean	SD	Mean	SD
*Na*	3.273	1.348	3.091	1.221	3.000	0.894	2.818	0.982
*Ho*	0.467	0.182	0.451	0.260	0.449	0.182	0.391	0.187
*He*	0.411	0.150	0.362	0.178	0.390	0.128	0.354	0.146
*PIC*	0.348	0.129	0.308	0.153	0.334	0.113	0.298	0.116
*Fis*	-0.132	0.123	-0.195	0.194	-0.137	0.151	-0.090	0.199
*HWD*	0.112		0.006^∗^		0.834		0.689	


*Na, number of alleles per locus; Ho, observed heterozygosity; He, expected heterozygosity; PIC, polymorphism information content; Fis, inbreeding coefficient. ^∗^indicated the probability of significant deviation from HWE based on all 11 SSR loci.*

## Discussion

### The WGS of *A. grahami* Provides a Useful Genetic Resource

If the initial discovery of a species can be treated as the first milestone for enabling people to know it, the WGS of a species would be another landmark to promote further applications. The advent of next-generation sequencing (NGS) has revolutionized genomics research by bringing the sequencing of entire genomes in a way of ever-increasing throughput and ever-decreasing cost (Van Dijk et al., 2014). This revolution has not only radically changed the paradigm of biological research, shifting it to a genome-wide scale, but also broadly opened up a new age in the whole biological sciences (Koboldt et al., 2013). Since the first completion of the human genome sequence in 2004, many WGS projects have been launched, such as the Genomes 10K Project (David et al., 2009), involving the sequencing of thousands or even millions of genomes (Van Dijk et al., 2014). The WGS is the basic genetic heritage for a species; WGS has ushered in a new era of investigation in biological sciences to the new sequenced species, allowing it to touch nearly every aspect of the biological enquiry (David et al., 2009).

Fishes account for over one-half of the world’s living species of vertebrates, exhibiting an incomparable diversity in their morphology, physiology, behavior, and ecological adaptations (Nelson et al., 2016). Fishes are also important food sources for humans, comprising 49.8 million tonnes of products, with an estimated first-sale value of US $99.2 billion in 2014 (FAO, 2016). The NGS-based WGS brings new opportunities to fish research and utilization; however, the current WGS projects on fishes, do not approach their diversity and application needs. The published genome data up to June 2018 was only available to 60 fish species (Hughes et al., 2018). These sequenced species are predominantly from the economically important fishes, such as Atlantic salmon (Davidson et al., 2010), common carp (Xu et al., 2014), and channel catfish (Liu et al., 2016); other sequenced species are either model organisms, including zebrafish (Howe et al., 2013) and medaka (Kasahara et al., 2007), or evolutionary interesting nodes, such as the coelacanth (Amemiya et al., 2013) and cavefishes (McGaugh et al., 2014).

In this study, we reported the WGS of a Yunnan-Guizhou plateau “3E” fish, *A. grahami*, which is a typical species with endangered, endemic, and economic status and priorities. Corresponding genome assembly of this teleost has been evaluated with good quality (Figure 1 and Table 2), and it was expected to provide a useful genetic resource for the further studies of this valuable fish.

### Inter-Subfamily Phylogenetic Relationships Within the Cyprinidae

In this study, we reconstructed for the first time the phylogenetic relationships within the Cyprinidae from a genomic viewpoint, combining the genomic data of *A. grahami* we obtained here with 18 other genomic and transcriptomic data of cyprinids that were downloaded from NCBI (Table 1). As we know, Cypriniformes is the largest monophyletic group of freshwater fishes in the world, with 4,000+ species recognized as well as 2,000+ species still awaiting for description (Mayden et al., 2009; Stout et al., 2016). Cyprinidae contains the vast majority of taxa in the Cypriniformes, and it is also the largest family of freshwater fishes on the earth (Nelson et al., 2016). Classification of subfamilies can facilitate the taxonomic, evolutionary and many other studies of this big group; however, the recognition of the subfamilies remains controversial in spite of some systematic studies. With 4,000+ recognized species, the ambition to reconstruct a tree of life at the species-level is largely impractical; however, using phylogeny-based subfamily classification could provide a simple but useful taxonomic system for broader studies.

A putative subfamily classification system, including 2 series and 10 subfamilies using skeletal characters, has been proposed (Chen et al., 1984). It was a fundamental framework for most of the ensuing taxonomic literature about Cyprinidae, such as in the books of “Fauna Sinica, Osteichthyes, Cypriniformes II & III” (Chen, 1998); and “Fishes of the World” (5th ed.), as well as some previous versions (Nelson et al., 2016). The previous classification (Chen et al., 1984) has been updated to a 12-subfamily system for the Cyprinidae, namely, Danioninae, Leuciscinae, Cultrinae, Xenocyprinae, Hypophthalmichthyinae, Cobioninae, Gobiobotinae, Acheilognathinae, Barbinae, Labeoninae, Schizothoracinae, and Cyprininae (Chen, 1998). This 12-subfamily classification has become one of the most useful and popular systems for subsequent studies (Chen, 2013), and due to its popularity, the inter-subfamily relationships under this classification system has also been testified by some of the molecular phylogenetic studies, mainly based on PCR-targeted DNA sequences (Wang et al., 2007, 2012).

The phylogenetic relationship in this study revealed three well-supported subclades of Cyprinidae (Figure 2). The subfamily Danioninae (herein as Clade I) was resolved as the basal-most subfamily within the Cyprinidae, which is consistent with some previous molecular phylogenetic studies (Gilles et al., 2001; Wang et al., 2007) but disagrees with some others (Chen and Mayden, 2009; Wang et al., 2012). Morphologically, Danioninae is a large assemblage containing mostly taxa unaccommodated by the other subfamilies (Wang et al., 2007). The sister group relationship of Clade II and III, in line with most of the previous studies based on PCR-targeted DNA sequences, supported two well-accepted major lineages within Cyprinidae, namely, barbeled cyprinines (herein as Clade II) and (usually) non-barbeled leuciscines (herein as Clade III, Wang et al., 2012). It was also largely consistent with the two series classification – the fundamental framework of (Chen et al., 1984) based on skeletal characters – except for the position of *Tinca*. Clade II was recovered with a relationship of [Labeoninae, (Schizothoracinae, (Cyprininae, Barbinae))], which was largely consistent with most previous studies based on more species but less sequence lengths (Wang et al., 2007, 2012), and this subclade has now been suggested to be a named Cyprininae (see review in Yang et al., 2015). Clade III was comprised of the species usually called as “the Endemic Clade of East Asian Cyprinidae” (Tao et al., 2010), even though the inter-group relationships were controversial based on previous PCR-targeted DNA sequences (Wang et al., 2007, 2012). Based on genome-level sequences used in this study, Clade III was recovered in a relationship of [(*Tinca*, (Acheilognathinae, Gobioninae)), ((Leuciscinae, Hypophthalmichthyinae), (Xenocyprinae, Cultrinae))]. Two sister group relationships among Clade III, the Acheilognathinae + Gobioninae, and the Xenocyprinae + Cultrinae, were broadly consistent with most of the previous studies; however, recovering *Tinca* as the sister group of other Leuciscinae from some other studies (Wang et al., 2007, 2012; Stout et al., 2016) was not supported in this study. *Tinca* has long been treated as *Incertae sedis* from both morphological and molecular studies (Wang et al., 2007). Due to its controversial phylogenetic position, the monotypic genus *Tinca* has been frequently suggested to be an independent subfamily as Tincinae (Wang et al., 2012; Stout et al., 2016).

The classification of subfamilies in the Cyprinidae and the subgroups embodied in each subfamily have varied among different studies, which has been inevitable in the progress toward the ultimate tree of life among 4,000+ cyprinids. During this process, many taxonomic levels, such as series, lineages, subfamilies, and tribes, were proposed to designate newly recognized groups (Yang et al., 2015; Stout et al., 2016); however, these complicated terms make the phylogenetic relationships of Cyprinidae inaccessible for most people without in-depth knowledge of this group. The phylogenetic relationship revealed in this study (Figure 2), in spite of the limited number of species included, is expected to provide a simple but useful framework of the inter-subfamily phylogeny of Cyprinidae.

### Historical Relationship Between *A. grahami* and Its Habitat Fuxian Lake

As one of the Yunnan-Guizhou plateau lakes, Fuxian Lake is the sole habitat of *A. grahami*. Interestingly, the species exhibits many special biological characters, which were believed to be a result of adaptation along with the long-term formation of Fuxian Lake (Yang, 1992). Fuxian Lake, similar to most of the other Yunnan-Guizhou plateau lakes, is a kind of rift lake that formed and evolved under long, periodic and complex tectonic events during the rising of Qinghai-Tibet plateau (Zhu et al., 1989).

From the evidence of lake sediments, we know that the Fuxian Lake was formed by fault-subsidence tectonics in the late Tertiary, and then sustained from pond to basin since late Pliocene (*ca*. 3.0–3.4 Ma). It experienced a large paleo-Fuxian Lake period in the late Pleistocene to Holocene (*ca*. 0.126–0.012 Ma), where the superficial area was approximately 1.6-fold greater and the surface elevation was 30–40 m higher than the lake at present (Zhu et al., 1989). Afterward, the lake body rapidly sunk and the mountains around gradually lifted, which finally shaped Fuxian Lake to be the second deepest lake in China, with an extreme depth at over 150 m and average depth at about 87 m. Along with the process of deepening, Fuxian lake has also been undergoing a copiotrophic to oligotrophic transformation (Yang, 1994). In summary, there are three periods during the development of Fuxian Lake (Figure 3): (I) lacus formation period since late Pliocene (*ca*. 3 Ma), (II) large lake period since late Pleistocene (*ca*. 0.1 Ma), and (III) a deepening period of the lake accompanied by oligotrophic development since early Holocene (*ca*. 0.012 Ma).

Interestingly, the population demography of *A. grahami* matched well with the three periods during the development of Fuxian Lake (Figure 3). The population of *A. grahami* maintained a relatively stable level at the early period (0.6–3 Ma), which would reflect the long time of the lacus formation since late Pliocene (*ca*. 3 Ma, in Period I). During this time, the ancestors of *A. grahami* colonized the lake and shifted gradually from lotic to lentic habitats. The population increase of *A. grahami* since 0.6 Ma would possibly be a response of the expansion of Fuxian Lake. When the fish reached the maximal population size (0.03–0.04 Ma), the lake was also had its largest ponding area (*ca*. 0.1 Ma, in Period II). A similar pattern was also detected by us in another adjacent Yunnan-Guizhou plateau lake, Dianchi Lake, when the endemic fish, *S. grahami*, exhibited a noteworthy population expansion congruent with a period when the paleo-Dianchi Lake had a three times larger area (Yang et al., 2016). In considering of the similar patterns between *A. grahami* in Fuxian Lake and *S. grahami* in Dianchi Lake, range expansion served as a crucial factor in increasing the population sizes of plateau endemic fishes, and *vice versa*. In *A. grahami*, the later shrinking and deepening of Fuxian Lake (*ca*. 0.012, Period III) might be the key reason for its population declining after the maximal population size (0.03–0.04 Ma). The oligotrophizing along with the deepening of Fuxian Lake would, synchronously and substantially, accelerate the speed of its population decline afterward.

### SSR Development and Utilization for Genetic Diversity Analysis

Molecular markers have been widely used to study the genetic diversity of a species. Because of the abundantly polymorphic, selectively neutral, highly repeatable, and unambiguously genotyping, SSR is one of the most useful molecular markers that can easily explore and apply in this post-genomic area. Compared to the traditionally expensive, time-consuming and labor-intensive in construction of the enriched libraries, identifying SSR markers based on high-throughput sequencing is much faster and more cost-effective (Liu et al., 2017). Identification of SSR markers provided valuable resources for further studies of each newly sequenced taxon (Stoll et al., 2017).

In this study, we identified 33,836 SSR loci of *A. grahami* after genomic searching under five criteria, which can serve as a SSR resource pool for studies on this species (Supplementary Table S6). We designed a three-step approach, namely, PCR, polymorphism and parameters evaluation (Figure 4), to assess this identified SSR resource pool by randomly selected 50 SSR loci for primer design and marker screening. After three steps evaluating and filtering, we retained 11 optimized SSR markers that can be used for a preliminary genetic diversity analysis (Supplementary Tables S8, S9). The *PIC* of each marker usually reveals the general diversity in the genetic analysis of a species. According to the *PIC* values of the 11 SSR markers in four populations (30 samples in each population), the average *PIC* value was calculated to be 0.322 among these four artificially cultivated populations (Table 3), which indicated the general genetic diversity of *A. grahami* was reasonably informative (Botstein et al., 1980).

Maintenance of genetic diversity is the major objective of most projects for conservation and utilization, so that population can face environmental challenges in the future and can respond to long-term selection, either natural or artificial for traits of economic and cultural interest (Sharma et al., 2016). From the perspective of conservation, reintroduction is the most popular technique for endangered species to re-establish populations within their historic range (IUCN, 1998). However, success of such projects largely depends on the correspondingly long-term management for the genetic diversity, population structure, levels of inbreeding and other relevant parameters (Tollington et al., 2013). As an endangered fish that has undergone drastic population decline in these decades, reintroduction of *A. grahami* to the Fuxian Lake has become the major way to re-establish its wild populations. Therefore, the artificial cultivated populations from fish breeding farms have been the main sources for the present and future wild populations. Based on the four artificial cultivated populations, we revealed that the general genetic diversity of *A. grahami* was moderate, and the inbreeding level within each of the four populations was relatively low (Table 3). It would suggest that the genetic diversity of *A. grahami* at present is not necessarily a cause for pessimism; however, a whole picture of its genetic diversity and population structure based on a broader sample coverage has yet to be uncovered.

In summary, besides the new assembled genome resource, the identified 33,836 SSR loci provided another useful genetic resource for long-term explorations of this “3E” species. Especially, the 11 optimized SSR loci screened from this study will provide practical genetic tools for further near-term genetic and conservation studies.

## Data Availability

This Whole Genome Shotgun project has been deposited at DDBJ/ENA/GenBank under the accession RJVU00000000 with a BioProject ID of PRJNA477399. The version described in this paper is version RJVU01000000.

## Author Contributions

JXY, WJ, QS, and LC conceived the project and designed the scientific objectives. WJ, XP, YZ, XW, KY, CS, and QL collected and prepared the fish samples. YQ, YL, CB, JL, XY, JC, and JLY conducted bioinformatics analysis. WJ and YZ performed the SSR development and experiments. WJ, YQ, and XP prepared the manuscript. QS, JXY, and LC revised the manuscript. All authors have read and approved the final manuscript.

## Conflict of Interest Statement

The authors declare that the research was conducted in the absence of any commercial or financial relationships that could be construed as a potential conflict of interest.

## Footnotes

1 http://pgrc.ipk-gatersleben.de/misa/misa.html
